# Neuronal knockdown of *Cullin3* as a *Drosophila* model of autism spectrum disorder

**DOI:** 10.1038/s41598-024-51657-9

**Published:** 2024-01-17

**Authors:** Samantha J. Tener, Zhi Lin, Scarlet J. Park, Kairaluchi Oraedu, Matthew Ulgherait, Emily Van Beek, Andrés Martínez-Muñiz, Meghan Pantalia, Jared A. Gatto, Julia Volpi, Nicholas Stavropoulos, William W. Ja, Julie C. Canman, Mimi Shirasu-Hiza

**Affiliations:** 1 Department of Genetics and Development, Columbia University Irving Medical Center, New York, NY 10032 USA; 2 Department of Neuroscience, The Herbert Wertheim UF Scripps Institute for Biomedical Innovation and Technology, Jupiter, FL 33458 USA; 3 Waksman Institute, Rutgers University, Piscataway, NJ 08854 USA; 4 Department of Pathology and Cell Biology, Columbia University Irving Medical Center, New York, NY 10032 USA

**Keywords:** Behavioural genetics, Autism spectrum disorders

## Abstract

Mutations in *Cullin-3* (*Cul3*), a conserved gene encoding a ubiquitin ligase, are strongly associated with autism spectrum disorder (ASD). Here, we characterize ASD-related pathologies caused by neuron-specific *Cul3* knockdown in *Drosophila*. We confirmed that neuronal *Cul3* knockdown causes short sleep, paralleling sleep disturbances in ASD. Because sleep defects and ASD are linked to metabolic dysregulation, we tested the starvation response of neuronal *Cul3* knockdown flies; they starved faster and had lower triacylglyceride levels than controls, suggesting defects in metabolic homeostasis. ASD is also characterized by increased biomarkers of oxidative stress; we found that neuronal *Cul3* knockdown increased sensitivity to hyperoxia, an exogenous oxidative stress. Additional hallmarks of ASD are deficits in social interactions and learning. Using a courtship suppression assay that measures social interactions and memory of prior courtship, we found that neuronal *Cul3* knockdown reduced courtship and learning compared to controls. Finally, we found that neuronal *Cul3* depletion alters the anatomy of the mushroom body, a brain region required for memory and sleep. Taken together, the ASD-related phenotypes of neuronal *Cul3* knockdown flies establish these flies as a genetic model to study molecular and cellular mechanisms underlying ASD pathology, including metabolic and oxidative stress dysregulation and neurodevelopment.

## Introduction

Autism spectrum disorder (ASD) is a neurodevelopmental condition associated with dysfunctions in social communication, executive function and cognition, sleep, and metabolism^1^. Recent estimates find that the prevalence of ASD is one in 44 children^2^ and that ASD is approximately 83% heritable^3^. Large-scale genomic sequencing over the past decade has identified more than 100 risk genes and genomic regions associated with the development of ASD^4–9^, but the identification of these genetic risk factors has yet to have a significant impact on the clinical treatment of ASD^10,11^. As these genetic risk factors span a variety of potential mechanisms contributing to ASD pathology, it is crucial that various genetic models of ASD are generated and explored to advance our understanding of how ASD may arise from multiple mechanisms.

Genomic sequencing studies have implicated de novo mutations in *Cullin-3* (*Cul3*) as causative factors for ASD^9,12–19^. Based on these studies, the Simons Foundation Autism Research Initiative (SFARI) gives *Cul3* a SFARI Gene Score of one^20^, signifying it as a high confidence autism risk gene. *Cul3* encodes a member of the Cullin protein family and acts as an E3 ubiquitin ligase, assembling with adaptor proteins that recruit specific protein substrates for ubiquitination^21–26^. *Cul3* is evolutionarily conserved from fruit flies to humans, with 71.67% sequence identity. *Drosophila* shares approximately 75% of disease-causing genes with humans and offers a wide variety of relevant assays and powerful, publicly available genetic tools^27–29^. Thus, *Drosophila melanogaster* is a potentially advantageous model system for studying ASD pathophysiology.

Because of this close association with ASD, *Cul3* mutants have previously been used to study protein degradation, brain development, and social behaviors in mice^30–37^. Cul3 has also surfaced in work regarding sleep, locomotion, and neurodevelopment in *C. elegans*^38,39^*.* In fruit flies, neuron-specific knockdown of *Cul3* has been studied primarily in the context of neuronal development and sleep^40–43^. A conserved, putative Cul3 adaptor protein, Insomniac (Inc), was shown to be important for the proper development of sleep-regulatory neurons in the fruit fly brain^44,45^. Because sleep disturbances are common in autism^46–48^, these findings suggest a mechanism by which *Cul3* lesions may cause a core symptom of ASD. It is not clear whether other symptoms and physiologies associated with ASD can also be observed in flies with neuronal *Cul3* knockdown.

Here, we performed a broad characterization of RNAi-mediated *Cul3* neuronal-specific knockdown as a potential genetic model for the study of ASD in fruit flies. We first validated the knockdown and the short-sleep phenotype. We then found that these flies were dysregulated in both metabolic and oxidative stress homeostasis, as indicated by increased sensitivity to starvation and hyperoxia. Neuronal *Cul3* knockdown flies also had fewer triacylglyceride stores than controls. Through a courtship suppression assay, we found that neuronal *Cul3* knockdown flies exhibited significantly less courtship behavior and a learning and memory deficit. Immunostaining revealed that *Cul3* neuronal knockdown exhibited alterations in the structure of the mushroom body, a brain region associated with memory and sleep. Thus, neuronal knockdown of *Cul3* in fruit flies recapitulates multiple phenotypes of ASD, including defects in sleep, metabolism, social interactions, cognition, and brain development, presenting a powerful model to dissect the underlying mechanisms by which this genetic risk factor might contribute to ASD.

## Results

### Validating the neuronal knockdown of *Cul3* and sleep phenotype

**Figure 1:**
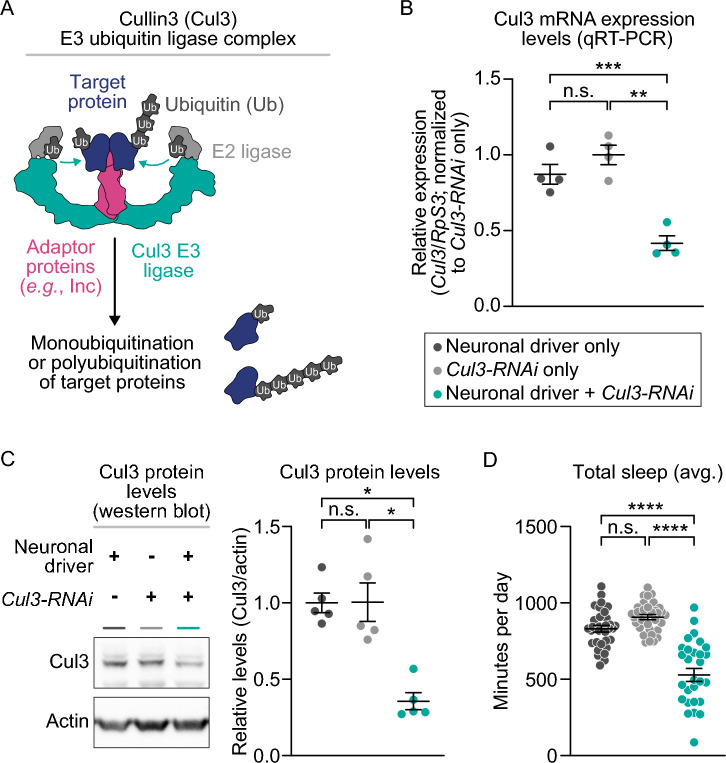
Validation of *Cul3* neuronal knockdown and sleep phenotype. (**A**) Schematic of the Cul3 protein, an E3 ligase, in the E3 ubiquitin ligase complex that targets proteins from degradation by the proteosome. (**B**) Neuronal *Cul3* knockdown flies exhibited less *Cul3* expression than controls by qRT-PCR (p = 0.0036 Gal4 control, p = 0.0010 UAS control, n = number of biological replicates, each containing 30 fly heads). (**C**) Western blot showing that neuronal *Cul3-RNAi* flies have reduced Cul3 protein compared to controls (p = 0.0112 Gal4 control, p = 0.0486 UAS control, n = number of biological replicates, each containing 10 fly heads). (**D**) Relative to controls (gray), flies with neuronal knockdown of *Cul3* (teal) exhibited fewer average total minutes of sleep per day (p < 0.0001, each data point represents a single fly). P-values were obtained by Brown-Forsythe and Welch ANOVA test (**B**,**D**) and Kruskal–Wallis test (**C**). Averages are shown with error bars representing SEM.

*Cul3* encodes a core component of a multiprotein E3 ubiquitin ligase complex that targets specific proteins for ubiquitination (Fig. 1A). To knock down *Cul3* expression, we performed RNAi depletion (*UAS-Cul3-RNAi*) using a neuron-specific Gal4 expression driver (*elav-GAL4*). We used 7- to 10-day old males for all assays, unless otherwise noted. To validate the neuronal knockdown of *Cul3*, we assessed *Cul3* mRNA levels in male fly heads using quantitative real-time PCR (qRT-PCR). We found that neuronal *Cul3* RNAi reduced *Cul3* expression by 58% relative to controls containing the *RNAi* construct alone and 52% relative to controls containing the *Gal4* driver alone (Fig. 1B). We then showed by Western blot analysis that neuronal *Cul3* RNAi led to a 65% reduction in Cul3 protein compared to controls containing the *RNAi* construct alone and 64% relative to controls containing the *Gal4* driver alone (Fig. 1C). We further confirmed that these flies exhibited a short-sleep phenotype, as previously described^40,41^ (Fig. 1D). Cul3 RNAi expressed with *elav-GAL4* reduced sleep by 42% and 36% respectively relative to controls containing either the RNAi construct or the *Gal4* driver.

### Neuronal knockdown of *Cul3* shortened survival under starvation

**Figure 2:**
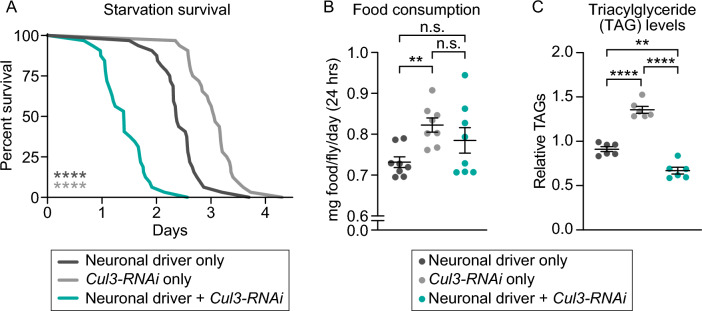
Neuronal *Cul3* knockdown flies exhibited starvation sensitivity and lower TAG levels but no difference in food consumption. (**A**) Flies with neuronal knockdown of *Cul3* (teal) died faster on starvation media (1% agar) than controls (gray) (*p* < 0.0001, each line represents 32 flies). (**B**) Neuronal knockdown of *Cul3* flies (teal) ate the same amount of food relative to control flies (gray) (*Gal4* control, *p* = 0.3646; *UAS* control, p = 0.6578; each data point represents an average of 4–5 flies). (**C**) Neuronal knockdown of *Cul3* flies (teal) had a significantly lower amount of triacylglycerides (TAGs) compared to control flies (*Gal4* control, *p* = 0.0018; *UAS* control, *p* < 0.0001; each data point represents an average of 20 flies). *p*-values were obtained by log-rank test (**A**) and Brown-Forsythe and Welch ANOVA test (**B**,**C**). Averages are shown, with error bars representing SEM.

Because both sleep deprivation^49–51^ and ASD^52–55^ have been linked to metabolic dysregulation, we tested whether *Cul3* neuronal knockdown flies exhibited metabolic differences. We performed a starvation survival assay and found that neuronal knockdown of *Cul3* significantly shortened survival time under starvation compared to either control (Fig. 2A), suggesting that neuronal *Cul3* reduction perturbs metabolic homeostasis. We next characterized the feeding behavior of these flies, using food labeled with a radioactive tracer (see Methods). We found that flies with neuronal knockdown of *Cul3* consumed similar amounts of food relative to controls, although one parental control ate significantly more than the other (Fig. 2B). This result suggests that feeding behavior does not contribute to the metabolic differences caused by neuronal *Cul3* knockdown. To further understand the starvation sensitivity phenotype, we measured levels of triacylglycerides (TAGs), the main fat storage molecules of both humans and *Drosophila* . Since neuronal *Cul3* knockdown flies were more susceptible to starvation, we hypothesized that they would have lower levels of TAGs relative to controls. Indeed, we found that TAG levels in *Cul3* knockdown flies were significantly lower than controls (Fig. 2C). Thus, insufficient TAG stores may contribute to the starvation sensitivity of neuronal *Cul3* knockdown flies, suggesting that lipid metabolism is dysregulated in these flies.

### Neuronal knockdown of *Cul3* conferred sensitivity to hyperoxia, an induced oxidative stress.

**Figure 3:**
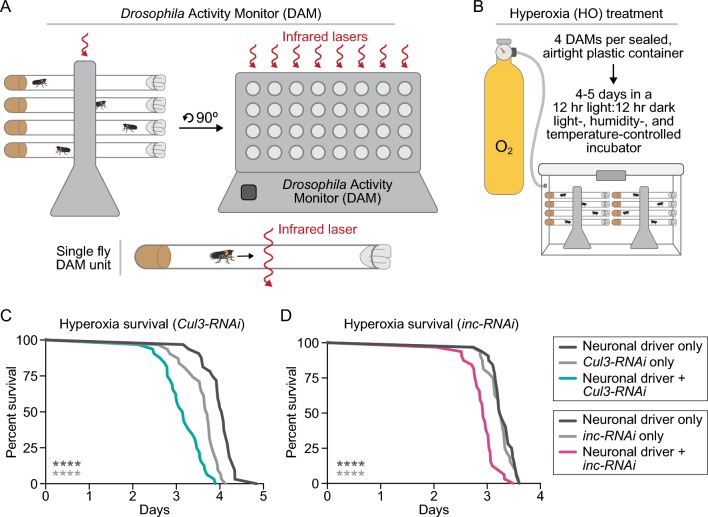
Flies with neuronal knockdown of *Cul3* or *inc* have increased sensitivity to hyperoxia. (**A**) Schematic of *Drosophila* Activity Monitors (DAMs). (**B**) Schematic of the set-up to apply hyperoxia, or 100% oxygen, to flies in DAMs. (**C**) Flies with neuronal knockdown of *Cul3* (teal) died faster in hyperoxia than controls (gray) (*p* < 0.0001). (**D**) Flies with neuronal knockdown of *inc* (pink) died faster in hyperoxia than controls (gray) (*p* < 0.0001). Each line represents 32 flies; p-values were obtained by log-rank test (**C**,**D**).

Biomarkers of oxidative stress are reported to be increased in ASD^56–58^. We previously found that short-sleeping flies, including those with neuronal knockdown of *Cul3* or *inc*, are more sensitive to an induced oxidative stress, such as hydrogen peroxide feeding or paraquat injection^42^. Because these previous methods of inducing oxidative stress are laborious and potentially variable across individual flies, we sought to develop a more consistent and efficient method of applying oxidative stress. To assess sensitivity to oxidative stress, we applied 100% oxygen (hereafter, "hyperoxia")^59^ to flies in DAMs contained within airtight chambers and monitored their time until death, using terminal loss of activity as a proxy for death (Fig. 3A,B). Using this method, we found that neuronal knockdown of either *Cul3* (Fig. 3C) or *inc* (Fig. 3D) significantly reduced survival time in hyperoxia with respect to controls. This result confirms our previous findings that neuronal knockdown of *Cul3* and *inc* lead to increased sensitivity to oxidative stress, including acute oxidative stress via exogenous hyperoxia.

### Neuronal knockdown of *Cul3* caused a courtship defect, as well as a learning and memory defect

ASD is associated with motor difficulties and impairments, such as hypotonia, motor apraxia and motor delay^60–62^. To assess motor function in neuronal *Cul3* knockdown flies, we assayed their climbing ability but found no difference in climbing ability relative to controls (Fig. S2).

**Figure 4:**
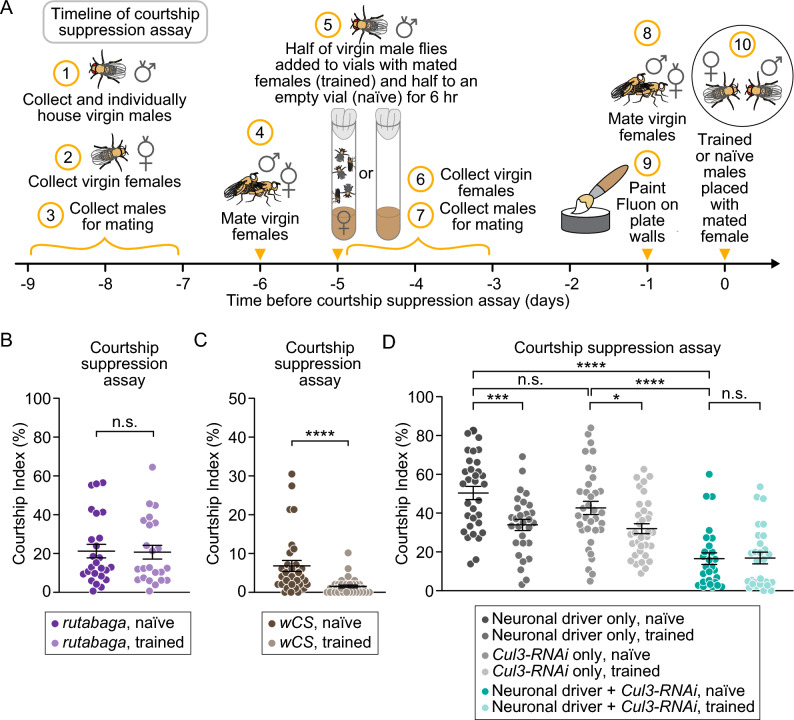
Flies with neuronal knockdown of *Cul3* exhibited a learning and memory deficit and less courtship behavior. (**A**) Schematic depicting the timeline of our courtship suppression assay, used to assess both courtship behavior and learning and memory. (**B**) Trained *rutabaga* mutants (light purple) did not differ in courtship index compared to naïve *rutabaga* mutants (purple) (p = 0.8022). (**C**) Trained *wCS* flies (tan) had a significantly higher courtship index than naïve *wCS* flies (brown) (*p* < 0.0001). (**D**) Trained neuronal *Cul3* knockdown flies (light teal) did not differ in courtship index as compared to naïve neuronal *Cul3* knockdown flies (teal) (*p* > 0.9999), while trained controls had significantly lower courtship indices than naïve controls (*Gal4* control, *p* = 0.0005; *UAS* control, *p* = 0.0152). Flies with neuronal knockdown of *Cul3* (teal) exhibited a lower courtship index than controls (gray) (*p* < 0.0001). Each point represents an individual fly; *p*-values were obtained by Mann–Whitney test (**B**,**C**; neuronal *Cul3* knockdown, **D**), Kruskal–Wallis test (**D**, all naïve groups compared), and Welch’s t test (controls, **D**). Averages are shown with error bars representing SEM.

Individuals with ASD typically exhibit altered social interactions and often have cognitive impairments related to learning and memory^63–65^. To test the effects of neuronal knockdown of *Cul3* on both social interactions and memory, we used the well-established courtship suppression assay (described in “Methods”, Fig. 4A)^66^. In brief, male *Drosophila* exhibit highly stereotyped behaviors to court female *Drosophila*; these behaviors can be videotaped and quantified. Here we used a combination of FlyTracker and JAABA software, together with a machine learning algorithm, for automated quantitation of male courtship behavior (see “Methods”)^67,68^. Courtship behavior by naïve males provides a measure of basal social interaction, whereas courtship in experienced (trained) males is also dependent on learning and memory. Males trained with sexually unreceptive females that have mated in the preceding 24 h learn to expect sexual rejection and are less likely to exhibit courtship behavior in their next encounter with a female^66^.

To test learning and memory, 2- to 4-day-old virgin males of each genotype were split into two groups and housed for 6 h either alone (naïve condition), or with a sexually unreceptive female (trained condition). After 5 days, all males were introduced to a sexually unreceptive female and recorded for 10 min to track and quantify their courtship behavior, as described in the Methods. In the case of normal learning and memory, trained flies exhibit reduced courtship due to memory of their previously rejected courtship attempts. For mutants with learning and memory defects, such as *rutabaga* (*rut*)^69^, which lack function for the enzyme adenylyl cyclase required for synaptic plasticity, trained males will court at the same level as naïve males (Fig. 4B). In contrast, *white Canton-S* (*wCS*) males were able to learn and remember previous experiences of sexual rejection even though they have low courtship indices due to vision defects^70,71^ (Fig. 4C). Thus, the courtship suppression assay is a robust measure of both basal courtship behavior (seen with naïve males) and learning and memory (seen with trained males).

To assess basal courtship behavior, we compared courtship in naïve neuronal *Cul3* knockdown males and naïve controls. We found that naïve males with neuronal knockdown of *Cul3* had a significantly lower courtship index relative to controls (Fig. 4D). This result indicates that neuronal knockdown of *Cul3* reduces courtship behavior, a critical social behavior in fruit flies. When we used this assay to compare courtship of naïve and trained neuronal *Cul3* knockdown males, we found that there was no difference in courtship (Fig. 4D), in contrast to trained control males which courted significantly less than their naïve siblings. This result suggests that flies with neuronal knockdown of *Cul3* have a learning and memory defect.

### Neuronal *Cul3* knockdown altered mushroom body anatomy

**Figure 5:**
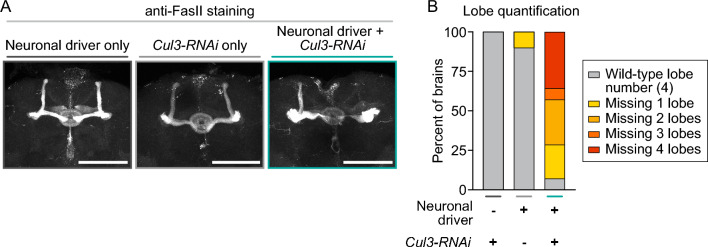
Neuronal *Cul3* knockdown alters mushroom body anatomy. (**A**) Adult brains stained with anti-FasII antibody are shown for neuronal driver control (*elav-Gal4*, left), RNAi construct control (*UAS-Cul3-RNAi/* +, center), and neuronal knockdown of *Cul3* (*elav-Gal4; UAS-Cul3-RNAi/* +, right) genotypes. For *Cul3* knockdown panel (right), note missing vertical lobe and attenuated medial lobe in left hemisphere and attenuated medial lobe in right hemisphere. Scale bars 100 μM. (**B**) Summary data for controls and *Cul3* knockdown brains. Number of αβ lobes missing entirely is indicated.

Autism is characterized by altered nervous system development^72^, and many ASD risk genes have developmental functions within the brain^73^. To assess whether deficits in memory and sleep caused by reduced neuronal *Cul3* activity might reflect anatomical changes in relevant brain regions, we examined the mushroom body, a structure required for memory and sleep^74–76^. We stained adult brains with an antibody recognizing Fasciclin II (FasII), which prominently marks the axons of mushroom body αβ neurons^77^. Control brains bearing *elav-Gal4* or *UAS-Cul3-RNAi* alone exhibited normal αβ projections that bifurcated into vertical and medial lobes in each hemisphere (Fig. 5A,B). In contrast, neuronal *Cul3* RNAi caused severe abnormalities in αβ axons, with ~ 93% of brains missing at least one lobe and ~ 36% of brains lacking αβ projections entirely (Fig. 5A,B). The loss of αβ axons was associated with enlarged FasII^+^ clusters, suggesting the accumulation of axons that failed to bifurcate (Fig. 5A,B and Fig. S3). In some brains, αβ projections were present but attenuated, and other brains exhibited morphological irregularities in the ellipsoid body, also marked by FasII (Fig. S3). These results indicate that neuronal *Cul3* activity is essential for the development and anatomy of the mushroom body, a structure important for memory and sleep.

## Discussion

*Cul3* mutations have been associated with autism spectrum disorder (ASD). In this work, we establish neuronal *Cul3* knockdown flies as a model system for the study of ASD. These flies share a variety of behavioral and physiological phenotypes that are associated with ASD in humans. We confirmed that neuronal *Cul3* knockdown flies are short sleeping, found that they exhibited increased sensitivity to both starvation and hyperoxia, and discovered that they exhibited decreased courtship behavior and a learning and memory defect. We also found that reduced Cul3 activity in neurons altered the structure of the mushroom body, a brain region vital for memory and sleep. Thus, neuronal *Cul3* reduction in *Drosophila* may serve as a useful model to elucidate mechanisms underlying behavioral and metabolic differences in ASD.

As a ubiquitin ligase, Cul3 binds dozens of adaptors and is likely to ubiquitinate hundreds of substrates^78–80^. This may help to explain why a range of behavioral and physiological phenotypes including sleep, metabolism, oxidative stress response, learning and memory, and social behaviors like courtship are all impacted by the neuronal knockdown of *Cul3* in flies and are implicated in ASD. While some specific targets of Cul3 have been identified^81–83^, a systematic investigation of proteins ubiquitinated by Cul3 may provide insight into how this variety of phenotypes in ASD and *Cul3* mutants is connected.

While intellectual disabilities and social behaviors are aspects of ASD frequently studied in humans and model systems, our analysis of *Cul3* neuronal knockdown flies also revealed two more unusual phenotypes: increased sensitivity to both starvation and hyperoxia, an induced oxidative stress. These findings suggest that reduced *Cul3* activity in neurons alters metabolic and oxidative stress homeostasis, both of which are associated with ASD in humans^52–58^. Reactive oxygen species, the main propagators of oxidative stress in a cell, are generated by mitochondria during oxidative phosphorylation. Because oxidative phosphorylation is the main mechanism for cellular ATP production, changes in metabolism will directly impact cellular oxidative stress levels. This begs the question of whether neuronal *Cul3* knockdown leads to perturbations in metabolism that subsequently cause an increase in oxidative stress, or whether these two phenotypes arise separately. We found that flies with neuronal knockdown of *Cul3* had significantly lower triacylglyceride stores, which may explain their sensitivity to starvation and ultimately suggests lipid metabolic dysregulation in these flies. This finding is consistent with studies that have observed lipid dysregulation as well as elevated levels of lipid peroxidation, a form of lipid oxidative damage, that could contribute further to lipid dysregulation in ASD^56,57,84^. Further study may reveal other aspects of metabolism that are altered by neuronal *Cul3* knockdown and whether such metabolic differences increase oxidative stress, establishing these as either related or separate pathologies in ASD.

Although many ASD risk genes are known to impact neuronal development, how specific ASD-associated mutations alter the development and function of discrete brain circuits is not well understood. We found that reduced *Cul3* activity in neurons altered projections of the mushroom body, a brain region essential for sleep and memory. Because neuronal *Cul3* knockdown also caused defects in sleep and memory, one simple hypothesis is that these phenotypes arise from developmental alterations of mushroom body neurons. Manipulations of *Cul3* within the mushroom body and other neuronal subpopulations could test this hypothesis and might enable *Cul3* phenotypes, including metabolic dysregulation and susceptibility to oxidative stress, to be mapped with greater resolution within the brain.

Our findings and earlier studies suggest that *Cul3* impacts neuronal development by multiple mechanisms. Homozygous *Cul3* mutant clones within the mushroom body were found to alter axonal and dendritic development of α′β′ and γ neurons, but have relatively few effects on αβ neurons^43^. The anatomical defects in αβ neurons caused by neuronal *Cul3* RNAi suggest that Cul3 may impact neuronal development through additional, cell non-autonomous mechanisms. The latter may be particularly relevant to autism associated with heterozygous *Cul3* mutations, which would be expected to broadly decrease *Cul3* activity throughout the brain. Additional effects of *Cul3* on neurogenesis, neuronal migration, and synaptic development have been observed in *Cul3* mutant mice^85–88^. Whether these phenotypes are conserved in flies remains to be addressed.

While *Cul3* likely contributes to ASD phenotypes through multiple adaptors and substrates, previous work on Inc, a putative Cul3 adaptor, has suggested their coordinated function during brain development^45^. Loss of *inc* function in developing neurons underlies sleep dysregulation in adult *inc* mutants, and *inc* mutants also exhibit alterations of mushroom body neurons similar to those caused by *Cul3* knockdown. It is unknown whether *Cul3* function during development or adulthood underlies the metabolic, oxidative stress, courtship, and learning and memory phenotypes caused by neuronal *Cul3* knockdown. Future studies utilizing a conditional neuronal knockdown of *Cul3*, specific to development or adulthood, may reveal the temporal contributions of *Cul3* in ASD-related behaviors and physiologies.

Our findings highlight neuronal knockdown of Cul3 in *Drosophila* as a useful model to further investigate the mechanisms linking ASD to sleep disturbances, metabolic dysregulation, oxidative stress response, learning and memory, and social deficits. We also establish exposure to 100% oxygen as a simple method to induce hyperoxia and study oxidative stress response in an ASD fly model. As the pathways underlying these various behaviors and physiologies are highly conserved between *Drosophila* and humans, we anticipate that further study of this *Cul3* fly model will be valuable for understanding the pathology of ASD.

## Methods

### Fly strains and rearing conditions

All flies were raised on glucose food (Archon) in a temperature- (25 °C) and humidity- (65%) controlled incubator with a 12-h light–dark cycle. 7- to 10-day-old males were used for all experiments, unless otherwise noted.

The following flies were used to manipulate *inc* and *Cul3* as described previously^41,42^: *UAS-inc-RNAi* (VDRC stock #18225), *elav*^C155^*-Gal4*, *UAS-Dicer-2* (Bloomington stock #24651), *UAS-Cul3-RNAi* (NIG stock #11861R-2), along with the isogenic *iso31* strain used for outcrossing. For neuronal *Cul3* knockdown experiments, the *UAS-Dicer-2* line (Bloomington stock #24651) was crossed into the *elav*^C155^*-Gal4* line. Parental controls used for experiments were obtained by crossing expression driver and RNAi construct lines to the outcrossed wild-type line for heterozygous controls, accounting for differences in phenotypes affected by genetic background. *rutabaga* mutants (*rut*^*1*^) were acquired from David Schneider.

### Sleep analysis

7- to 10-day-old male flies entrained on a 12:12 light–dark (LD) cycle were placed in individual 5 mm plastic tubes containing food. Tubes were placed in TriKinetics *Drosophila* Activity Monitors (DAMs) to record their locomotor activity for 2 days in 12:12 LD. Beam break data were grouped into 1 min bins using DAM File Scan, and pySolo (Python-based software) was used to analyze sleep architecture and waking activity. Sleep was defined as a period of at least 5 min of inactivity^89^.

### Hyperoxia assay

7- to 10-day-old male flies entrained on a 12:12 light–dark cycle were placed in individual 5 mm plastic tubes containing food and loaded into *Drosophila* Activity Monitors. The monitors were placed in airtight chambers (Kent Scientific VetFlo™ Low Cost Induction Chamber) filled with 100% oxygen starting at ZT0 (“lights on”) until all flies were dead. Time of death was determined by complete loss of movement.

### Starvation assay

7- to 10-day-old male flies entrained on a 12:12 light–dark cycle were placed in individual 5 mm plastic tubes containing 1% agar and loaded into *Drosophila* Activity Monitors. Time of death was determined by complete loss of movement.

### Survival curves

Survival curves for starvation assays and hyperoxia assays are all plotted as Kaplan–Meier graphs. Log-rank analysis was performed using GraphPad Prism. All experiments were performed with a minimum of three independent trials and yielded statistically similar results, except where noted. Graphs and *p*-values in figures are from representative trials.

### qRT-PCR

7 to 10-day-old male flies entrained to 12:12 LD were frozen at -80ºC and heads were separated by vortexing and collected on ice. RNA was extracted from 30 fly heads for each of 4 biological replicates per genotype with TRIzol (Invitrogen) following the manufacturer’s protocol. Samples were treated with DNaseI (Invitrogen), then heat inactivated. cDNA was synthesized by Revertaid First Strand cDNA Synthesis Kit (Thermo Scientific). PowerUp SYBR Mastermix (Applied Biosystems) was used to perform qRT-PCR using a CFXConnect thermal cycler (BioRad). Primer efficiency and relative quantification of transcripts were determined using a standard curve of serial diluted cDNA. Transcripts were normalized using *ribosomal protein S3* (*RPS3*) as a reference gene.

Primer sequences:

*Cul3*-fwd-ATGCTACTTTTGTCGCCCATCGC

*Cul3*-rev-CTGGGTTATCCTTGGTTTATCCTGGCCT

*RPS3*-fwd-CGAACCTTCCGATTTCCAAGAAACGC

*RPS3*-rev-ACGACGGACGGCCAGTCCTCC

### Feeding assay

As previously described^90^, approximately 7-day-old flies were fed ^32^P-labeled Archon food for 24 h. Scintillation counts of accumulated ^32^P were quantified for an average of 4–5 flies per sample to estimate the amount of food consumed.

### Triacylglyceride measurement using thin-layer chromatography

Each biological replicate sample was comprised of 20 male flies. Samples were homogenized in a 2:1 chloroform:ethanol solution, then centrifuged for 10 min at 4 °C. 10 μL of each sample was loaded on a TLC plate (Sigma Aldrich Silica gel on TLC-PET foils 99577-25EA) and separated by polarity using a solvent solution (70 mL n-hexane, 30 mL diethyl ether, and 1 mL acetic acid). A reference sample of 20 wildtype female flies in 2:1 chloroform:ethanol solution was included on each plate. Plates were stained with 0.2% amido black (NAPHTHOL BLUE BLACK, Sigma Product N-3393-100G) in 1 M NaCl and imaged with an iBright™ FL1500 Imaging System. Images were analyzed using FIJI, subtracting individual lane background for each sample and normalizing to the reference sample.

### Western blot analysis

Dissected head lysates of female flies (10 flies/sample) were separated by SDS-PAGE using standard procedures. Membranes were probed with antibodies raised against the conserved C-terminal region of human Cul3 at 1:1000 (Cell Signaling, 2759 s) and (HRP)-conjugated monoclonal mouse anti-actin antibody at 1:5000 (Sigma-Aldrich, A3854). Rabbit primary antibody was detected using HRP-conjugated anti-rabbit IgG antibodies at 1:2000 (Cell Signaling, 7074). ECL chemiluminescence reagent (Pierce) was used to visualize horseradish peroxidase activity and detected by CCD camera iBright 1500 (Thermo-Fisher). FIJI gel analysis tool was used for densitometric quantification. A minimum of 5 independent samples for each condition were used for statistical analysis.

### Climbing assay

Assessment of climbing ability was performed as previously described^91^. Eight vials were transferred to an empty standard 23 mm × 95 mm plastic vial and then gently tapped to the bottom. The number of flies that reached the top quarter of the vial within 20 s were then scored as climbing.

### Courtship suppression assay

Virgin males were collected within 6 h of eclosion and stored in individual vials. 3–4 days post-collection, individual males were transferred to training vials (15 mm × 65 mm). For the “trained” condition, a sexually unreceptive *wCS* female (mated within the last 24 h) was added to the training vial with the virgin male; for the “naïve” condition, no female was added to the training vial with the male. Six hours following conditioning, males were isolated and housed in individual vials for another 5 days (memory consolidation period). During testing, trained and naïve males were placed with an unreceptive *wCS* female in 16 mm diameter chambers without food and videotaped for 10 min to assess courtship memory.

The CalTech FlyTracker and JAABA softwares were used to automatically quantify the Courtship Index (CI), or percent of frames spent courting in a 10 min period, for each male. The Caltech FlyTracker automatically generates data for each video frame such as the location of the male and female, the angle of the wings, the velocity of the flies, etc. We visually inspected each video to ensure tracking accuracy; if a video had tracking errors in > 5% of frames, we discarded the video. We trained the machine learning algorithm (JAABA) to quantify the wing extension, chasing, and attempted mounting behaviors for courtship frame by frame based on the data from the Caltech FlyTracker. Since two or more behaviors can co-occur simultaneously (such as wing extension and chasing in courtship), it is typical in the field to use a hierarchical code that would not double count frames as “courtship” when computing total courtship levels. Our courtship code gives greatest priority to wing extension, followed by chasing and attempted mounting; that is, if wing extension and chasing co-occur in a video frame, JAABA annotates the frame for wing extension only.

### Immunostaining and imaging

Brains from adult male progeny were dissected in ice-cold PBS and fixed in 4% paraformaldehyde in PBS for 20 min at room temperature on a nutator. All subsequent incubations and washes were performed at room temperature and on a nutator unless specified otherwise. Fixative was removed and brains were washed in PBS containing 0.3% Triton X-100 (PBST) three times for 15 min each. Samples were then incubated in blocking solution, containing 5% normal donkey serum in PBST, for 30 min, followed by incubation for 2 days at 4 °C in primary antibody cocktail, prepared in blocking solution and containing mouse anti-Fasciclin II (1:50, DSHB, 1D4) and rat anti-elav (1:50, DSHB, 7E8A10) antibodies. Primary antibody cocktail was removed and samples were washed in PBST three times for 15 min each. Samples were then incubated for 2 days at 4 °C in secondary antibody cocktail prepared in blocking solution and containing Alexa 488 donkey anti-mouse (1:1000, Life Technologies, A21202) or Alexa 647 donkey anti-mouse (1:1000, Life Technologies, A31571) and Rhodamine Red-X donkey anti-rat (1:1000, Jackson Immunoresearch, 712-295-153) antibodies; samples were wrapped in foil to protect them from light for this and subsequent steps. Samples were washed three times in PBST for 15 min each and subsequently incubated for 30 min in 0.8 μg/ml DAPI in PBST. Samples were rinsed three times in PBST and mounted in Vectashield (Vector labs) on slides and covered with #1.5 coverslips, using bridges prepared from #2 coverslips. Samples were imaged on Leica Stellaris or SP8 microscopes using a 20 × oil immersion objective to capture z-stacks at 512 × 512 or 1024 × 1024 resolution. The same gain settings were used for experimental and control samples. Maximal projections were generated in Fiji/Image J. The presence or absence of vertical and horizontal mushroom body lobes was scored in a non-blinded manner.

### Statistical analysis

We assessed the normality of our data using the D’Agostino-Pearson omnibus normality test and the Shapiro–Wilk normality test, which have good power properties over a wide range of distributions^92^. For datasets that passed both normality tests, we used the unpaired Student’s t-test with Welch’s correction when comparing two groups and the one-way ANOVA (Dunn’s multiple comparisons) when comparing three or more groups. For data sets that failed either one of the normality tests, we used the Mann–Whitney U test when comparing two groups and the Kruskal–Wallis test with Dunn’s post hoc test when comparing three or more groups. Significance is expressed as *p*values (n.s., *p* > 0.05; *p < 0.05; **p < 0.01; ***p < 0.001; ****p < 0.0001).

### Supplementary Information

Supplementary Figure 1.

Supplementary Figure 2.

Supplementary Figure 3.

Supplementary Legends.

## Data Availability

The authors declare that all data supporting the findings of this study are available, including replicate experiments, and will be made available upon reasonable request to the corresponding author, Dr. Mimi Shirasu-Hiza.
