# Supplementary figures and images for: Neuronal knockdown of Cullin3 as a Drosophila model of autism spectrum disorder
Source: Sci Rep. 2024 Jan 17;14:1541. doi: 10.1038/s41598-024-51657-9 (PMC10794434; doi:10.1038/s41598-024-51657-9)

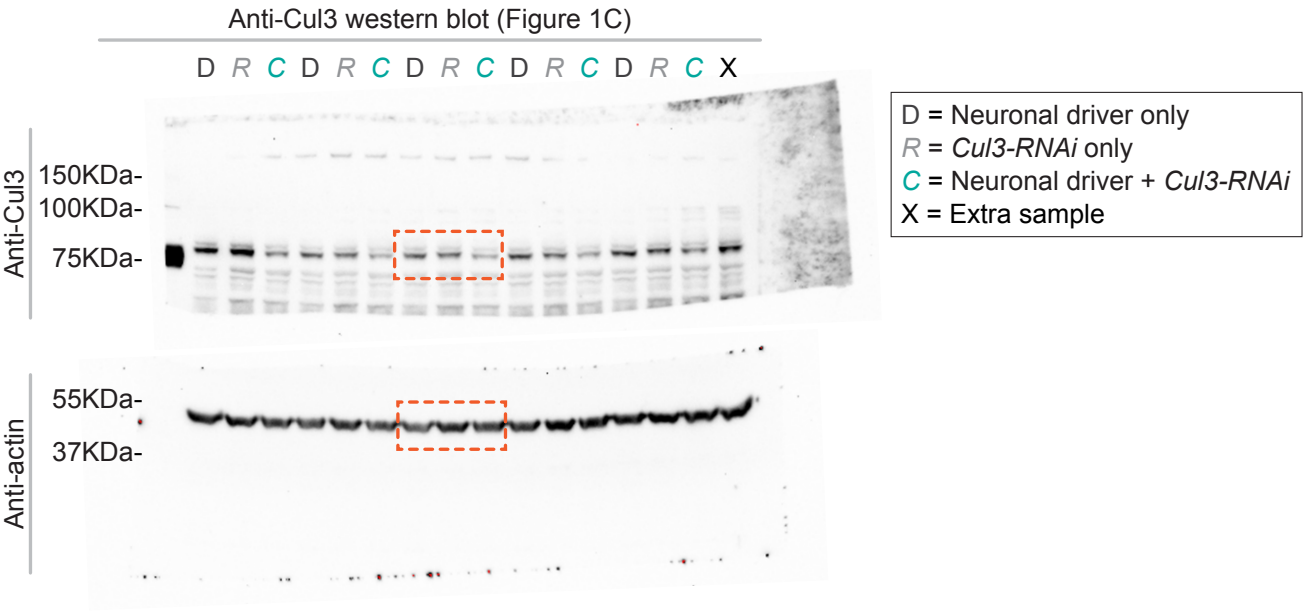

Supplement: Supplementary file 1 — Supplementary Figure 1. [file 41598_2024_51657_MOESM1_ESM.pdf]

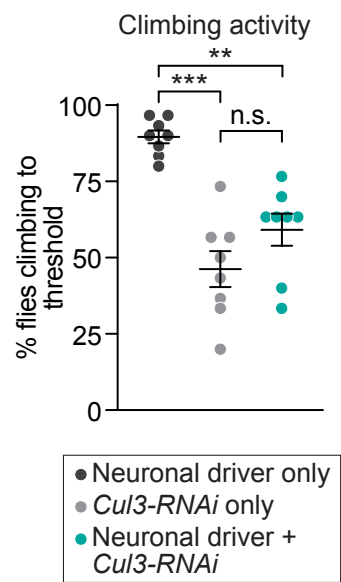

Supplement: Supplementary file 2 — Supplementary Figure 2. [file 41598_2024_51657_MOESM2_ESM.pdf]

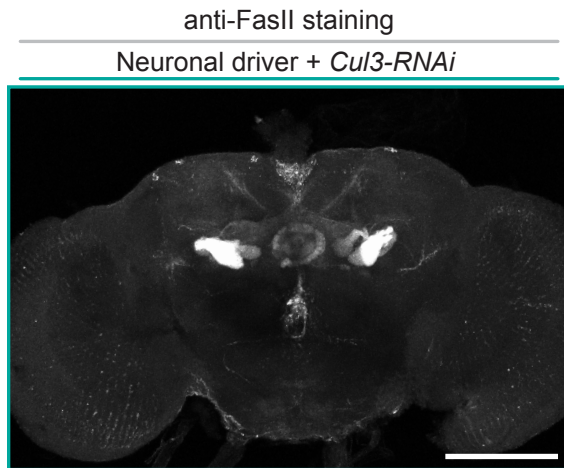

Supplement: Supplementary file 3 — Supplementary Figure 3. [file 41598_2024_51657_MOESM3_ESM.pdf]
