# Supplementary material for: Neuronal knockdown of Cullin3 as a Drosophila model of autism spectrum disorder
Source: Sci Rep. 2024 Jan 17;14:1541. doi: 10.1038/s41598-024-51657-9 (PMC10794434; doi:10.1038/s41598-024-51657-9)
Supplement: Supplementary file 4 — Supplementary Legends. [file 41598_2024_51657_MOESM4_ESM.docx]

**Figure S1. Flies with neuronal knockdown of *Cul3* had decreased levels of Cul3 protein.** Whole western blot showing that *Cul3* neuronal knockdown flies had decreased levels of Cul3 protein; red dashed line indicates section shown in Fig. 1C.

**Figure S2. Flies with neuronal knockdown of *Cul3* did not exhibit a climbing defect.** Relative to controls (gray), flies with neuronal knockdown of *Cul3* (teal) did not have a difference in climbing activity (*Gal4* control, *p*=0.0013; *UAS* control, *p* =0.3102; (n=8 vials of 10 flies/genotype). Each point represents an average of 10 flies; *p*-values were obtained by Brown-Forsythe and Welch ANOVA test. Averages are shown with error bars representing SEM.

**Figure S3. Defects in the mushroom body and ellipsoid body caused by neuronal Cul3 depletion.** Neuronal knockdown of Cul3 (*elav-Gal4; UAS-Cul3-RNAi/+)* brain exhibiting absence of normal αβ projections, enlarged FasII^+^ foci, presence of γ lobes, and aberrant ellipsoid body. Scale bar, 100 μM.
